# Analytical Performance of a Multiplex Real-Time PCR Assay Using TaqMan Probes for Quantification of *Trypanosoma cruzi* Satellite DNA in Blood Samples

**DOI:** 10.1371/journal.pntd.0002000

**Published:** 2013-01-17

**Authors:** Tomas Duffy, Carolina I. Cura, Juan C. Ramirez, Teresa Abate, Nelly M. Cayo, Rudy Parrado, Zoraida Diaz Bello, Elsa Velazquez, Arturo Muñoz-Calderon, Natalia A. Juiz, Joaquín Basile, Lineth Garcia, Adelina Riarte, Julio R. Nasser, Susana B. Ocampo, Zaida E. Yadon, Faustino Torrico, Belkisyole Alarcón de Noya, Isabela Ribeiro, Alejandro G. Schijman

**Affiliations:** 1 Grupo de Biología Molecular de la Enfermedad de Chagas, Instituto de Investigaciones en Ingeniería Genética y Biología Molecular “Dr. Héctor N. Torres” (INGEBI-CONICET), Buenos Aires, Argentina; 2 Instituto de Medicina Tropical, Universidad Central de Venezuela, Caracas, Venezuela; 3 Instituto de Biología de la Altura, Universidad Nacional de Jujuy, Jujuy, Argentina; 4 Universidad San Simón, Cochabamba, Bolivia; 5 Instituto Nacional de Parasitología “Dr. Mario Fatala Chaben”, ANLIS, Buenos Aires, Argentina; 6 Laboratorio de Química Biológica, Facultad de Ciencias Naturales, Universidad Nacional de Salta, Salta, Argentina; 7 Pan-American Health Organization, Washington, D.C., United States of America; 8 Drugs and Neglected Diseases Initiative, Genève, Switzerland; United States Food and Drug Administration, United States of America

## Abstract

**Background:**

The analytical validation of sensitive, accurate and standardized Real-Time PCR methods for *Trypanosoma cruzi* quantification is crucial to provide a reliable laboratory tool for diagnosis of recent infections as well as for monitoring treatment efficacy.

**Methods/Principal Findings:**

We have standardized and validated a multiplex Real-Time quantitative PCR assay (qPCR) based on TaqMan technology, aiming to quantify *T. cruzi* satellite DNA as well as an internal amplification control (IAC) in a single-tube reaction. IAC amplification allows rule out false negative PCR results due to inhibitory substances or loss of DNA during sample processing. The assay has a limit of detection (LOD) of 0.70 parasite equivalents/mL and a limit of quantification (LOQ) of 1.53 parasite equivalents/mL starting from non-boiled Guanidine EDTA blood spiked with *T. cruzi* CL-Brener stock. The method was evaluated with blood samples collected from Chagas disease patients experiencing different clinical stages and epidemiological scenarios: 1- Sixteen Venezuelan patients from an outbreak of oral transmission, 2- Sixty three Bolivian patients suffering chronic Chagas disease, 3- Thirty four Argentinean cases with chronic Chagas disease, 4- Twenty seven newborns to seropositive mothers, 5- A seronegative receptor who got infected after transplantation with a cadaveric kidney explanted from an infected subject.

**Conclusions/Significance:**

The performing parameters of this assay encourage its application to early assessment of *T. cruzi* infection in cases in which serological methods are not informative, such as recent infections by oral contamination or congenital transmission or after transplantation with organs from seropositive donors, as well as for monitoring Chagas disease patients under etiological treatment.

## Author Summary

Chagas disease, caused by the parasite *Trypanosoma cruzi*, is endemic in several Latin American countries and still represents a major neglected tropical threat. It is transmitted to humans by blood-sucking triatomine bugs, congenital transmission, blood transfusion, organ transplantation and by consuming food and juice contaminated with the parasite. Tools for accurate diagnosis and surrogate markers of parasitological response to treatment remain key needs in the field. This study focused on the evaluation of a novel quantitative PCR assay for the diagnosis and follow-up of patients with Chagas disease, on the basis of international guidelines for analytical validation of molecular diagnostic methods. The method allows the simultaneous amplification of parasite satellite DNA sequence and a heterologous internal amplification control that permits rule out false negative results due to inhibitory substances or loss of DNA during sample processing. It was evaluated in peripheral blood samples from acute and chronic patients as well as in umbilical cord blood samples from newborns to seropositive mothers. The performing characteristics of this assay position it as a promising candidate for application to clinical trials and kit developments.

## Introduction

Chagas disease, caused by the protozoan parasite *Trypanosoma cruzi* (*T. cruzi*), remains a major public health concern in 21 endemic countries of America, with an estimated prevalence of 8 million infected people [1].

The human disease occurs in two stages: an acute stage, which occurs shortly after an initial infection, and a chronic stage that develops over many years. Out of individuals at the chronic stage, 60–80% will never develop symptoms, while the remaining 20–40% will develop life-threatening heart and/or digestive disorders during their lifetime [1], [2].

Individuals from different endemic regions are infected with distinct parasite populations, recently classified into six Discrete Typing Units (DTUs), designated as *T. cruzi* I (TcI) to *T. cruzi* VI (TcVI) [3], initially defined as “sets of stocks that are genetically more related to each other than to any other stock and that are identifiable by common genetic, molecular or immunological markers” [4]. These DTUs are differently distributed in the endemic regions and transmission cycles and probably are differently involved in the clinical manifestations and severity of the disease [5], [6]. TcI is the major cause of Chagas disease in northern South America and Central America and prevails in wild cycles throughout the continent [6], whereas TcII, TcV and TcVI are predominant in the southern cone [7]–[10]. Moreover, remarkable intra-DTU variability has been observed within TcI, hence five groups of genotypes (TcIa to TcIe) have been proposed [11]–[16].

Current chemotherapies are more effective in recent infections than in chronic disease [17], being the serological conversion to negative the accepted criteria for cure, which usually occurs years after treatment, hampering the execution of clinical trials using novel drugs in chronically infected adult cohorts [18]. On the other hand, parasitological response to treatment is usually monitored by means of Strout, hemoculture or xenodiagnosis, which lack of sensitivity in the chronic phase [19].

In this context, the development of sensitive and accurate quantitative PCR (qPCR) strategies for *T. cruzi* quantification is crucial to provide a surrogate marker to assess treatment efficacy. A few real-time PCR strategies have been developed for detection of *T. cruzi* in Chagas disease patients [20]–[22]. Our group developed a SYBR-Green based qPCR strategy which used an internal amplification control (IAC) that was added to each blood sample prior to DNA extraction [22]. Although this meant an improvement in qPCR for Chagas disease, amplification of *T. cruzi* and IAC targets had to be done in separate tubes. Accordingly, we developed and standardized a multiplex qPCR strategy based on TaqMan technology, aiming to quantify both *T. cruzi* and IAC DNAs in a single-tube multiplex reaction. This work presents the analytical validation and evaluation of this qPCR test in blood samples from Chagas disease patients under diverse clinical and epidemiological scenarios.

## Methods

### Ethics statement

The study was approved by the ethical committees of the participating institutions, namely, Comité de Bioética de la Provincia de Jujuy (CPBJ) and Comité de Bioética de la ANLIS “Dr Carlos G. Malbrán”, Ministerio de Salud, Argentina; Comité de Bioética de la Facultad de Medicina, Universidad Mayor de San Simón, Cochabamba, Bolivia; Comité de Bioética del Instituto de Medicina Tropical, Universidad Central de Venezuela, Caracas, Venezuela; following the principles expressed in the Declaration of Helsinki. Written informed consents were obtained from the adult patients and from parents/guardians on behalf of all newborns and children participants.

### Spiked blood samples

Seronegative human blood samples were spiked with cultured epimastigotes of Sylvio X10 and CL-Brener stocks (TcI and TcVI, respectively) and mixed with one volume of Guanidine Hidrochloride 6M, EDTA 0.2 M buffer, pH 8.00 (GE).

### Internal amplification control

A pZErO-2 recombinant plasmid containing an inserted sequence of *Arabidopsis thaliana* aquaporin was used as an heterologous extrinsic IAC [22]. The recombinant was gently provided by Dr Jorge Muschietti and coworkers (INGEBI-CONICET, Argentina). It was used to transform *Escherichia coli* bacteria in the presence of kanamicine to obtain plasmidic DNA after column extraction. For PCR purposes, the recombinant plasmid was linearized using the restriction enzyme Pst1.

### Patients and clinical specimens

The assay was evaluated in different groups of patients, as follows:

Group 1 (G1): Sixteen Venezuelan patients detected during the study of an outbreak of oral transmission of *T. cruzi* in an urban school in the Municipality of Chacao, Caracas, Venezuela [23]. All 16 patients were symptomatic, presenting facial edema, long lasting high fever and decay. Serological studies were positive on the basis of ELISA-IgM, ELISA-IgG, indirect hemagglutination test and lytic antibodies. The patients were treated with Benznidazole for one week plus three months with Nifurtimox and followed-up during two years after treatment. The qPCR assay was carried out at time of diagnosis, and 24 and 48 months after the end of treatment. Culture isolates obtained from one of these patients were genotyped as TcId (Diaz Bello Z et al., unpublished data). Five mL of peripheral blood samples were obtained for the analysis and immediately mixed with an equal volume of GE buffer, boiled during 15 min. and conserved at −20°C.

Group 2 (G2): Sixty three chronic Chagas disease patients from Bolivia (Chagas Epidemiological Network, Dr Faustino Torrico and DNDi, Dr Isabela Ribeiro). Ten mL of peripheral blood samples were obtained for the analysis and immediately mixed with an equal volume of GE and conserved at 4°C.

Group 3 (G3): Thirty four patients with chronic Chagas disease from Argentina admitted to a clinical trial entitled TRAENA (“Tratamiento en adultos”, Dr Adelina Riarte, unpublished data). Ten mL of peripheral blood samples were obtained and immediately mixed with an equal volume of GE, boiled during 15 min. and conserved at 4°C.

Group 4 (G4): Twenty seven out of 74 newborns to seropositive mothers delivered at Hospital Pablo Soria, San Salvador de Jujuy, Argentina from September 2011 to March 2012, were analyzed by qPCR. This province has been declared free of vectorial transmission [24]. Serodiagnosis of pregnant women was done by means of conventional serological methods. Newborns were tested by the microhematocrite test [25] and positive cases were treated with Benznidazole. Three out of the 74 newborns (4.0%) were positive by the microhematocrite method.

In eight newborns, 5 mL of umbilical cord blood was collected at delivery, in other 15 cases 1 mL of peripheral blood was withdrawn, and in four ones both umbilical and peripheral blood were collected. The umbilical cord was clamped, the segment was cleaned with a broad-range antiseptic product (Povidone-iodine, Phoenix Lab; Argentina) and 5 mL of blood was withdrawn from the end closer to the placenta. Samples were collected in tubes containing an equal volume of GE, boiled during 15 min. and stored at 4°C for DNA purification and PCR analysis.

Group 5 (G5): One seronegative patient (42 years old, man) that received on emergency a kidney transplant from a seropositive cadaveric donor followed up by Dr Roberta Lattes at the “ Instituto de Nefrología Buenos Aires”. Infection by *T. cruzi* was diagnosed by serological methods and Strout 121 days after transplantation and Benznidazole treatment was implemented during 60 days. Samples were treated with an equal volume of GE, boiled during 15 min. and stored at 4°C for DNA purification and PCR analysis.

### DNA extraction

Blood samples treated with GE (GEB) from G1, G2, G3 and G5 were processed using the High Pure PCR Template Preparation kit (Roche Diagnostics Corp., Indiana, USA): Five µL of linearized IAC (40 pg/µL) were added to 100 µL of binding solution in a clean tube and 300 µL of GEB (G2) or 200 or 300 µL of boiled GEB (G1 and G3/G5, respectively) were added and the mix was homogenized. This quantity of IAC was chosen because it renders a Ct value around 20, which is in the middle of the linear range of IAC amplification, as reported [22].

The solution was further mixed with 40 µL of proteinase K by vortexing during 15 sec., spinned down and incubated at 70°C for 10 min. in a dry thermo-block. After spin down, 100 µL of isopropanol were added, vortexed during 15 sec. and spinned down. Each sample was loaded into an extraction column placed into a 2 mL microtube. The content was centrifuged at 8000 rpm during 1 min. The extraction column was placed into a new collection tube. Inhibitors removing solution (500 µL) was added to each column and centrifuged as described before. The column was placed into a new tube. Washing solution (500 µL) was added to the column and centrifuged as described before. The column was placed into a new tube and the washing step was repeated. The column was placed into a 1.5 mL microtube and centrifuged at maximum speed for 10 sec. One hundred µL of pre-heated elution buffer were added to the column and centrifuged as previously described. The eluate was stored at −20°C for qPCR analysis. In order to build the standard curves for quantification of parasitic loads in G1, G2, G3 and G5 patients' specimens, DNA from spiked blood was prepared in the same way as reported for the clinical samples.

Three hundred µL of boiled GEB samples from G4 newborns were processed using the QIAamp DNA Mini Kit, after addition of 5 µL of linearized IAC (40 pg/µL) to the lysis buffer and processed as recommended by the manufacturer (Qiagen, USA). DNA from spiked blood used to build the respective standard curve for quantification was extracted as described for G4 samples.

### Multiplex real-time PCR standardization

On the basis of a previously reported TaqMan procedure for detection of *T. cruzi* satellite DNA [21] that showed high sensitivity and specificity in an international PCR study [26], we assayed the same *T. cruzi* primers and probe and designed a set of primers and probe for the IAC target (Table 1). The melting temperatures of IAC Fw and IAC Rv primers are similar to those of Cruzi 1 and Cruzi 2 primers (61.2°C, 60.9°C, 58.4°C and 59.5°C, respectively) using Oligo Calculator version 3.26 at http://www.basic.northwestern.edu/biotools/oligocalc.html.

**Table 1:** Sequences and concentrations of primers and probes used for the Multiplex Taqman qPCR assay.

Target	Oligonucleotide	Sequence	Final Concentration (µM)
*T. cruzi* satellite DNA	Cruzi 1	5′-ASTCGGCTGATCGTTTTCGA-3	0.75
	Cruzi 2	5′ -AATTCCTCCAAGCAGCGGATA-3	0.75
	Cruzi 3	5′ -Fam-CACACACTGGACACCAA-NFQ-MGB-3′	0.05
IAC	IAC Fw	5′ -ACCGTCATGGAACAGCACGTA-3′	0.1
	IAC Rv	5′ -CTCCCGCAACAAACCCTATAAAT-3′	0.1
	IAC Tq	5′ -VIC-AGCATCTGTTCTTGAAGGT-NFQ-MGB-3′	0.05

IAC: Internal amplification control. S: C/G.

The qPCR reactions were carried out with 5 µL of re-suspended DNA, using FastStart Universal Probe Master Mix (Roche Diagnostics GmbHCorp, Mannheim, Germany) in a final volume of 20 µL. Optimal cycling conditions were a first step of 10 min. at 95°C followed by 40 cycles at 95°C for 15 sec. and 58°C for 1 min. The amplifications were carried out in a Rotor-Gene 6000 (Corbett, UK) or in an Applied Biosystems (ABI 7500, USA) device. Standard curves were constructed with 1/10 and 1/2 serial dilutions of total DNA obtained from a GEB sample spiked with 10^5^ par. eq./mL of blood. TcI and TcVI based standard curves were used to quantify parasitic loads in G1 and in G2–G5 samples, respectively.

In order to evaluate the influence of the concentrations of IAC template, primers and probe in the efficiency of *T. cruzi* DNA amplification in the multiplex format, DNA extracts from samples carrying 0.5 to 750 par. eq./mL as well as samples without *T. cruzi* were amplified by both simplex qPCR (only *T. cruzi* primers and probe) and multiplex qPCR formats.

In order to assess the influence of *T. cruzi* load on the efficiency of IAC amplification in the multiplex format, *T. cruzi* DNA samples obtained to build the CL-Brener standard curve were amplified and the IAC was quantified. For this, a standard curve was built with DNA obtained from 300 µL of GEB spiked with 50 to 800 pg of linear IAC on duplicate as well as the PCR assay from each DNA lysate.

### Multiplex real-time PCR assay analytical performance

#### Terms

On the basis of the MICROVAL protocol [27], several key terms were defined in this study as follows: i) *Selectivity* is defined as a measure of the degree of response from target and non-target microorganisms and comprises inclusivity and exclusivity. *Inclusivity* is the ability of an alternative method (Real Time PCR in this case) to detect the target pathogen from different strains (Discrete Typing Units in this case), and *Exclusivity* is the lack of response from closely related but non-target strains (other Tripanosomatides in this case); ii)

#### Anticipated reportable range

A set of values of measurands for which the error of a measuring instrument is intended to lie within specified limits; iii) *Limit of detection (LOD)*: the smallest amount that the method can reliably detect to determine presence or absence of an analyte; iv) *Precision*: Closeness of agreement between independent test/measurement results obtained under stipulated conditions; v) *Limit of quantification (LOQ)*: The smallest amount the method can reliably measure quantitatively.

#### Inclusivity

The assay was evaluated with genomic DNA obtained from a panel of *T. cruzi* stocks belonging to the six different DTUs in concentrations ranging from 0.0625 to 10 fg/µL tested on duplicates: TcI [stocks K98 (spliced leader intergenic region based genotype TcIa), G (genotype TcId) and SE 9V (genotype TcIe)], [13]–[16]; TcII (stock Tu18), TcIII (stock M5361), TcIV (stock CanIII), TcV (stock PAH265) and TcVI (stock CL-Brener) [3].

Stocks K98 and CL Brener were grown at INGEBI. Strains PAH265, Tu18, CanIII and M5631 were kindly provided by Dr Patricio Diosque (INPE, Universidad Nacional de Salta, Argentina). The isolate SE 9V was kindly provided by Dr Aldo Solari (Fac. Medicina, Universidad de Chile, Santiago de Chile, Chile) and G was provided by Dr Jose Franco Da Silveira (EPM, Sao Paulo, Brazil).

#### Exclusivity

Serial dilutions of *Trypanosoma rangeli* and *Leishmania major*, *Leishmania mexicana* and *Leishmania amazonensis* purified DNA ranging from 1 to 1000 pg/µL were assayed on duplicates. *T. rangeli* DNA was kindly provided by Dr Juan David Ramirez and Dr Felipe Guhl (CIMPAT, Universidad de los Andres, Colombia) and *Leishmania* sp. DNA by Dr Paula Marcet (CDC, Atlanta, USA).

#### Anticipated reportable range

Cultured Sylvio X10 (TcId) and CL-Brener (TcVI) parasites were spiked into 10 mL of non-infected human blood, immediately mixed with an equal volume of GE, to obtain a panel of GEB samples spanning 10^5^ to 0.0625 par. eq./mL of blood. After DNA purification, each dilution was amplified on triplicate. Assigned versus measured values were converted to log_10_ par. eq./10 mL of blood and plotted for linear regression analysis.

#### Limit of detection

The LOD was calculated as the lowest parasitic load that gives ≥95% of PCR positive results, according to the NCCLS guidelines [28]. Due to the fact that many published *T. cruzi* PCR procedures used boiled GEB samples [26], the LOD was characterized from two panels of GEB samples spiked with the CL-Brener stock; one panel was boiled during 15 min. before preparing serial dilutions and the other one was diluted without prior boiling. For both panels, eight replicates from GEB dilutions containing 0.125, 0.25, 0.5 and 1 par. eq./mL of blood were purified and amplified during 5 consecutive days. The LOD was determined by Probit regression analysis (Probit Minitab 15 software, USA).

#### Precision

Precision experiments were performed with spiked GEB samples at concentrations of 0.5, 10 and 10^3^ par. eq./mL (0.69, 2 and 4 log_10_ par. eq./10 mL), assayed on duplicates during 20 consecutive experiments, one run per day, according to the NCCLS document EP5-A2 [29]. The estimates of within-device or within-laboratory precision standard deviations (St) were calculated using the formula S_t_ = [B^2^+(N−1)/N*S_r_^2^]^1/2^, being B the standard deviation of the daily means and S_r_ the estimate of repeatability standard deviation (within-run precision).

#### Limit of quantification

The LOQ was derived from a 20% threshold value for the coefficient of variation (CV) of measurements obtained in the precision experiments, following the recommendations of NCCLS document EP17-A [28]. Assuming an exponential decrease in CV, a curve for the relationship between CV and log_10_ par. eq./10 mL was fitted using SigmaPlot version 10.0 for Windows (SPSS, Chicago, IL).

### Quality controls for analysis of clinical specimens

A negative control and two positive controls containing different concentrations of *T. cruzi* DNA were included in every run: namely a high-positive control and a low-positive control near the lower limit of detection, as recommended [30].

### Statistics

The Tukey's criterion (boxplots) [31] was used to detect samples with outlier Ct values of IAC (Cts>75th percentile+1.5×interquartile distance of median Ct), which would indicate inhibition or material loss in samples from a same experiment/clinical group with n>10.

The bilateral t test was done to compare the IAC recovery between a) boiled and not boiled spiked GEB samples, b) umbilical and peripheral blood samples in G4 newborns, c) peripheral blood samples from G2 and G3 chronic cases after elimination of outlier samples, and d) samples processed using QIAamp versus Roche DNA extraction kits.

Values of p<0.05 were considered as significative. The software InfoStat 2012 (Infostat/Students version 2.0. Infostat/FCA Group. Córdoba's National University; Ed. Brujas, Córdoba, Argentina) was used for the analysis. Satterwait's correction was applied in cases of non-homogeneous variances.

## Results

### Standardization of the multiplex real-time PCR assay

We compared the qPCR positivity in 1 fg and 10 fg of purified DNA samples from cultured parasites of reference stock CL-Brener and in a panel of GEB samples from 18 chronic Chagas disease patients from Cochabamba, Bolivia using four different commercial Master Mixes developed for real-time PCR: namely TaqMan Fast Advanced Master Mix (Invitrogen, USA), FastStart Universal Probe Master Mix (Roche Diagnostics GmbHCorp, Mannheim, Germany), TaqMan Universal PCR Master Mix (Applied Biosystems, USA) and Multiplex PCR Kit, (Qiagen, USA). Each Master Mix was challenged with different combinations of Cruzi 1 and Cruzi 2 primers (0.25, 0.5, 0.75 and 1 µM) and Cruzi 3 TaqMan probe (50, 100, 200 and 400 nM) concentrations. A first experiment using purified *T. cruzi* DNA, allowed discarding TaqMan Fast Advanced Master Mix (Invitrogen) because it was incapable of detecting 10 fg of *T. cruzi* DNA. The remaining 3 Master Mixes were evaluated using 5 µL of DNA lysates obtained from the mentioned panel of GEB samples, out of which the FastStart Universal Probe Master Mix (Roche) gave 12 PCR positive results (66.67%), the Multiplex PCR Kit (Qiagen) gave 7 PCR positive results (38.89%, also positive with Fast-Start Universal Probe Master Mix) and the TaqMan Universal PCR Master Mix (Applied Biosystems) gave 3 PCR positive results (16.67%, also positive with the other Master Mixes). Accordingly, subsequent optimization and validation of the multiplex assay was carried out using FastStart Universal Probe Master Mix and the concentrations of primers and probes described in Table 1.

In multiplexed assays, IAC amplification must be limited to avoid competition with subsequent *T. cruzi* DNA amplification. Thus, we evaluated different concentrations of IAC Fw and Rv primers (0.06, 0.08, 0.1, 0.2 and 0.5 µM) and IAC TaqMan probe (50, 100, 200 and 400 nM) to obtain a limiting IAC amplification with high efficiency. Higher analytical sensitivity was achieved working with 0.75 µM *T. cruzi* primers, 0.1 µM IAC primers and 50 nM of *T. cruzi* and IAC TaqMan probes, using the FastStart Universal Probe Master Mix (Roche Diagnostics GmbHCorp, Mannheim, Germany). Similar Ct values for a panel of *T. cruzi* DNA concentrations were obtained when qPCR was carried out in simplex or multiplex formats, indicating that IAC template as well as IAC primers and probe did not interfere with the efficiency of parasite DNA amplification (data not shown). Moreover, *T. cruzi* DNA samples spanning 0.25 par. eq./mL to 10^5^ par. eq./mL did not interfere in the efficiency of IAC amplification, indicating no inhibition of the IAC in the presence of the tested parasite loads (data not shown).

### Internal amplification control

Amplification of IAC standard curve had an efficiency of 91.7% (y = −3.539x+19.831, R^2^ = 0.994; Figure S1). Besides, no significant differences in IAC amplification were obtained from 22 replicates of boiled and not boiled spiked GEB samples, giving mean Ct values of 19.13 (IC_95%_ [19.07–19.19]) and 19.04 (IC_95%_ [18.92–19.16]), respectively (p = 0.2204).

### Analytical performance of the multiplex real-time PCR assay

#### Selectivity

The Multiplex qPCR assay was challenged with parasite stocks belonging to the six DTUs. It detected 0.0625 fg/µL DNA from stocks representing TcIa, TcII, TcIII, TcV and TcVI; 0.25 fg/µL of DNA from TcId and TcIV stocks and 1 fg/µL of TcIe stock (Table 2).

**Table 2:** Inclusivity assay for *T. cruzi* DTUs.

	DTUs (Mean Ct)							
Conc. (fg/µL)	TcIa	TcId	TcIe	TcII	TcIII	TcIV	TcV	TcVI
**0.0625**	31.74	Undet.	Undet.	32.74^a^	28.69	Undet.	31.15	32.02^a^
**0.125**	31.03^a^	Undet.	Undet.	32.46	27.64	Undet.	31.17	35.32
**0.25**	30.62	38.06^a^	Undet.	30.42	27.14	37.21^a^	30.06	33.74
**1**	29.14	31.46	32.89^a^	28.88	24.33	32.32	28.25	30.02
**10**	26.22	28.13	31.98	25.13	23.98	29.76	25.88	27.93

Results are shown as mean Ct obtained from duplicates of each DNA concentration.

a Only one replicate was detected. TcIa: K98; TcId: G; TcIe: SE9V; TcII: Tu18; TcIII: M5361; TcIV: CanIII; TcV: PAH265; TcVI: CL Brener. Ct: threshold cycle; Undet.: not detectable.

The qPCR assay was challenged with serial dilutions of purified DNA from *T. rangeli* and *L. amazonensis*, *L. major* and *L. mexicana* stocks, ranging from 1 to 1000 pg/µL (Table 3). No amplification was observed from *Leishmania* sp. DNA but one of both replicates containing 10 and 100 pg/µL of *T. rangeli* DNA was qPCR positive, as well as both replicates from the highest tested concentration (Table 3).

**Table 3:** Exclusivity assay with other Trypanosomatids.

	Trypanosomatid (Mean Ct)			
Conc. (pg/µL)	*T. rangeli*	*L. major*	*L. mexicana*	*L. amazonensis*
**1**	Undet.	Undet.	Undet.	Undet.
**10**	36.29^a^	Undet.	Undet.	Undet.
**100**	32.96^a^	Undet.	Undet.	Undet.
**1000**	30.65	Undet.	Undet.	Undet.

Results are shown as mean Ct obtained from duplicates of each DNA concentration.

a Only one replicate was detected. Ct: threshold cycle; Undet.: not detectable.

#### Anticipated reportable range

**Figure 1:**
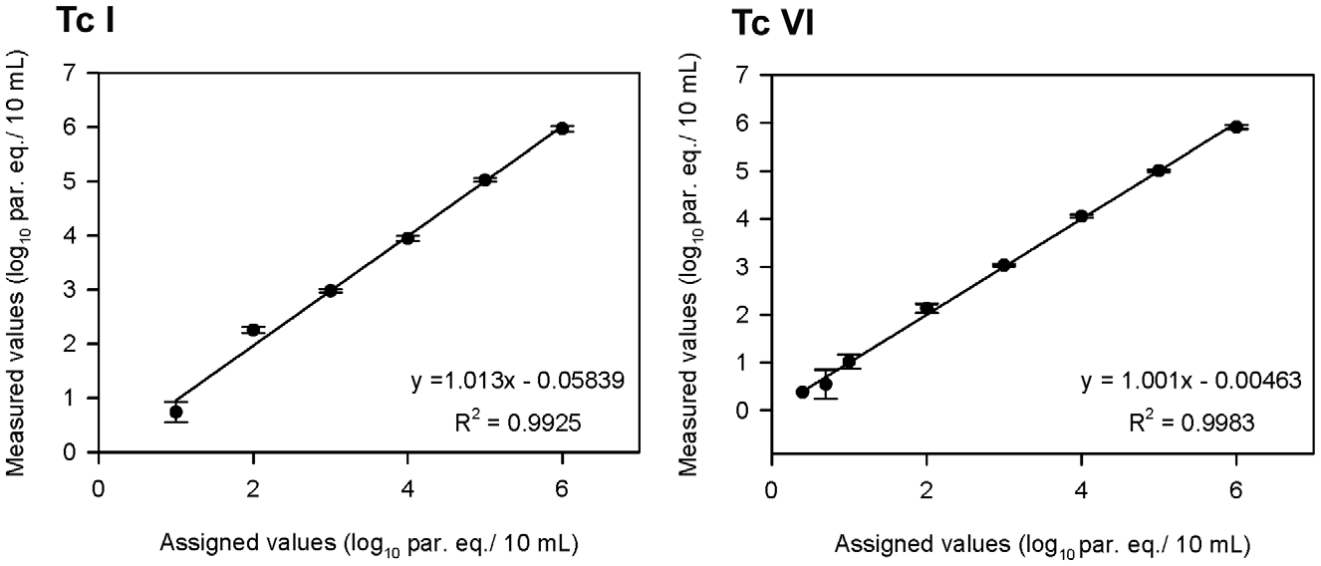
Anticipated reportable range and linearity of qPCR method. Multiplex TaqMan qPCR strategy was carried out with spiked GEB samples containing parasite stocks belonging to TcI and TcVI in ten concentrations spanning 10^6^ to 0.625 par. eq./10 mL, tested in triplicate. Assigned values were plotted on the *x* axis versus measured values (converted to log_10_) on the *y* axis using SigmaPlot 10.0 for Windows (SPSS, Chicago, IL). Linear regression analysis rendered the equation y = 1.013x+0.058 (R^2^ = 0.992) for TcI, and y = 1.001x+0.005 (R^2^ = 0.998) for TcVI.

The reportable range was calculated using spiked GEB samples containing serial dilutions of Silvio X10 (TcI) and CL Brener (TcVI) cultured epimastigotes. A linearity experiment was performed with a panel of 10 spiked GEB dilutions per parasite stock, spanning 10^5^ to 0.0625 par. eq./mL blood and tested in triplicate. Linear regression analysis gave the equation y = 1.013x+0.058 (R^2^ = 0.992) for TcI, and y = 1.001x+0.005 (R^2^ = 0.998) for TcVI. Thus, the reportable range was between 1 and 6 log_10_ par. eq./10 mL for the TcI stock and between 0.25 and 6 log_10_ par. eq./10 mL for the TcVI stock (Figure 1).***Limit of detection.*** Probit regression analysis showed LODs of 0.4619 (IC_95%_ [0.3645–0.6390]) and 0.6979 par. eq./mL (IC_95%_ [0.5396–1.012]) for boiled and non-boiled blood samples, respectively (p = 0.044).

#### Precision

The estimates of precision were calculated for 0.69, 2 and 4 log_10_ par. eq./10 mL of non-boiled GEB spiked with CL-Brener, equivalent to 0.5, 10 and 10^3^ par. eq./mL, respectively. Each dilution was assayed on duplicate during 20 consecutive days, one run per day (Table S1). The coefficients of variation of the precision estimates were 46.6, 6.00 and 1.72%, for 0.69, 2 and 4 log_10_ par. eq./10 mL, respectively (Table 4).

**Table 4:** Estimation of Precision of the qPCR assay.

Precision estimate	0.69 log_10_ par. eq./10 mL	2 log_10_ par. eq./10 mL	4 log_10_ par. eq./10 mL
**S_r_**	0.616	0.177	0.086
**B**	0.338	0.088	0.049
**N**	2	2	2
**Media**	1.183	2.549	4.516
**S_t_**	0.551	0.153	0.078
**CV%**	46.60	6.00	1.72

S_r_: estimate of repeatability standard deviation (within-run precision); B: standard deviation of the daily means; N: number of replicate analyses per run; S_t_: estimate of within-device or within-laboratory precision standard deviations (S_t_ = [B^2^+(N−1)/N*S_r_^2^]^1/2^); CV: coefficient of variation; log_10_ par. eq./10 mL: logarithmic values of parasite equivalents in 10 mL of blood.

#### Limit of quantification

**Figure 2:**
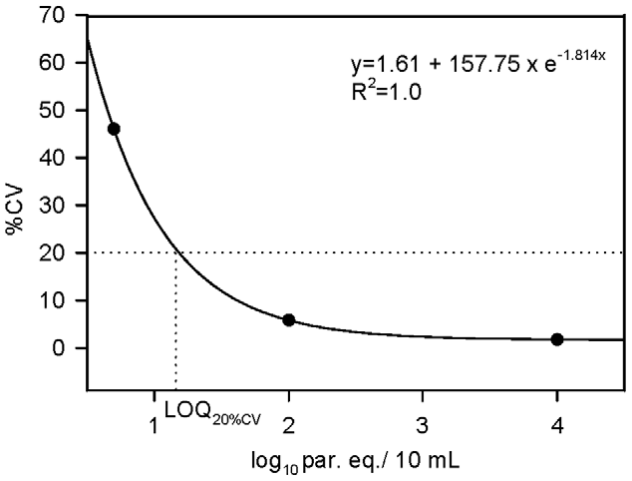
Estimation of the Limit of quantification of qPCR method. The LOQ was derived from a 20% threshold value for the coefficient of variation (CV) of measurements obtained in the precision experiments reported in Table 4. Linear least squares curve fit for relationship between CV and parasite concentration (log_10_ par. eq./10 mL) using SigmaPlot 10.0 for Windows (SPSS, Chicago, IL). The derivation of LOQ_20%CV_ is illustrated by dotted lines.

The LOQ was derived from a 20% threshold value of the CVs obtained in the precision experiments. Linear least squares regression for the equation y = y_0_+a×e^−bx^ resulted in the best fit (R^2^ = 1.0) for the variables y_0_ = 1.61, a = 157.75 and b = 1.814. Figure 2 displays the fitted curve and the derivation of LOQ_20%CV_. Based upon the derived equation, the absolute LOQ_20%CV_ was estimated as 1.185 log_10_ par. eq./10 mL, which corresponds to 1.531 CL Brener par. eq./mL of non-boiled GEB.

### Quantification of parasitic loads in Chagas disease patients

**Figure 3:**
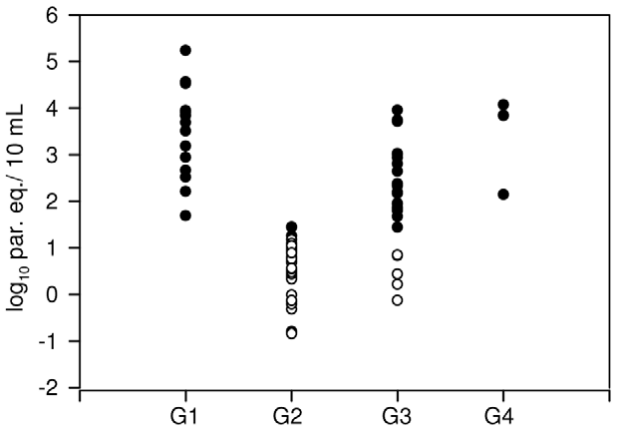
Distribution of parasitic loads in different patients' groups. Detectable qPCR findings obtained from peripheral blood samples of Chagas disease patients: G1, orally-acquired infected patients from Chacao, Venezuela (n = 14); G2, chronic Chagas disease patients from Cochabamba, Bolivia (n = 38); G3, chronic Chagas disease patients from endemic regions of Argentina (n = 26); G4, congenitally infected newborns to seropositive women (n = 3). LOQ: Limit of quantification. •: Quantifiable samples above LOQ, ○: Detectable samples below LOQ (1.185 log_10_ par. eq./10 mL).

The Multiplex qPCR test was carried out on blood samples from different groups of patients, namely Venezuelan patients infected by the oral route (G1, n = 16), chronic Chagas disease patients from Bolivia (G2, n = 63) and Argentina (G3, n = 34) and newborns to seropositive women (G4, n = 27); in the latter group, peripheral blood as well as cord blood samples were tested (Table 5 and Figure 3).

**Table 5:** Parasitic loads in Chagas disease clinical groups.

					(log_10_ par. eq./10 mL)		
Group	Procedence	Characteristics	Total	qPCR pos (%)	Median	Per 25	Per 75
**G1**	Venezuela	Oral Infection	16	14 (87.5%)	3.60	2.73	3.93
**G2**	Cochabamba	Chronic CD	63	38 (60.3%)	1.44	1.44	1.44
**G3**	Argentina	Chronic CD	34	26 (76.5%)	2.20	1.87	2.94
**G4**	North Argentina	Cong CD Newborns	3^a^	3 (100%)	3.84	2.99	3.95

CD: Chagas disease; Cong: Congenital; Pos: Positivity; Per: Percentile; par. eq./10 mL: parasite equivalents in 10 mL of blood.

a Three out of 27 newborns to seropositive mothers were diagnosed as congenitally infected by means of conventional diagnosis.

The proportion of qPCR positive results was 87.5% in G1, and 60.3 to 76.5% in G2 and G3, respectively (Table 5). In G4, only 3 out of the 27 newborns to seropositive mothers were qPCR positive, two cases were detected from the umbilical cord blood sample (case 1: *T. cruzi* Ct: 21.14, 3.84 log_10_ par. eq./10 mL, IAC Ct: 18.10 and case 2: *T. cruzi* Ct: 20.27, 4.07 log_10_ par. eq./10 mL, IAC Ct: 18.05) whereas the third one was detected from the peripheral blood sample (case 3, *T. cruzi* Ct: 27.51, 2.14 log_10_ par. eq./10 mL, IAC Ct: 19.42). These three cases were diagnosed as congenitally infected by means of the microhematocrite assay, thus, concordance between qPCR and microhematocrite was 100%.

The parasitic loads were heterogeneous in the studied populations, being highest in G4 and lowest in G2, in which only three out of the 38 qPCR positive samples were quantifiable (1.25, 1.44 and 1.45 log_10_ par. eq./10 mL blood), indicating in the majority of G2 patients very low parasitic loads, below the LOQ of the assay (Figure 3). On the other hand, the individual with highest parasitic load belonged to G1, presenting 5.23 log_10_ par. eq./10 mL blood, compatible with an acute infection (Figure 3).

In order to validate the above mentioned *T. cruzi* qPCR results on each clinical group on the basis of IAC recovery, the Tukey's criterion was applied to each group of tested specimens, allowing detection of outliers (Table 6). No outliers were obtained, except for a single blood sample from G2 (PCC 311, IAC Ct 19.20, Table 6).

**Table 6:** Estimation of IAC amplification in blood specimens from different clinical groups.

IAC	G1	G2	G3	G4	G4
Amplification	n = 16	n = 63	n = 35	Nb UCB	Nb PB
				n = 12	n = 19
**Median Ct**	19.31	18.20	18.72	19.97	19.24
**75th percentile**	20.11	18.43	19.01	21.00	19.86
**25th percentile**	18.37	18.01	18.44	19.10	18.88
**Threshold Ct for outlier values**	22.72	19.05^a^	19.86	23.86	21.32
**Media Ct**	19.24	18.15	18.70	20.22	19.20

a Sample PCC 331: qPCR positive, 0.69 log_10_ par. eq./mL, IAC Ct 19.20.

Nb: Newborn; UCB, umbilical cord blood; PB: peripheral blood.

Moreover, given that some groups of clinical specimens were processed using different DNA extraction kits, we compared the IAC recovery between samples extracted using the QIAamp DNA Mini Kit (Qiagen) with those using the High Pure PCR Template Preparation kit (Roche) (mean IAC-PCR Cts 19.60 vs 18.35, respectively, p<0,0001), showing higher recovery using the latter kit.

### Monitoring of acute infections and etiological treatment

**Figure 4:**
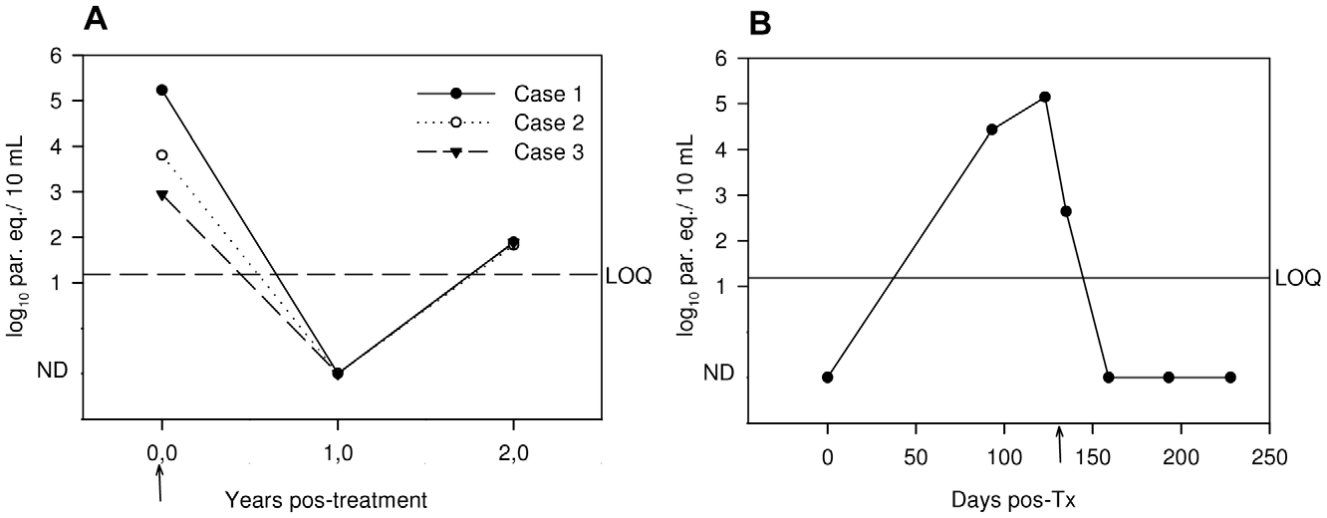
Follow-up of *T. cruzi* infected patients using qPCR. A. Follow-up of orally infected cases from Chacao, Caracas, Venezuela. Years pos-treatment (ys pos-T) are represented in the *x*-axis. Parasite equivalents (par. eq.) were estimated using a Silvio X-10 (TcI) calibration curve. Case 1- Pre-T: 5.23 log_10_ par. eq./10 mL; 2 ys pos-T: 1.88 log_10_ par. eq./10 mL. Case 2- Pre-T: 3.78 log_10_ par. eq./10 mL; 2 ys pos-T: 1.83 log_10_ par. eq./10 mL. Case 3- Pre-T: 2.94 log_10_ par. eq./10 mL; 2 ys pos-T: 1.88 log_10_ par. eq./10 mL. B. A 42 year-old seronegative man received kidney transplantation from a seropositive cadaveric donor. Progression of parasitic load after transplantation is shown as well as post-treatment follow-up. The quantification was estimated using a Cl-Brener (TcVI) calibration curve. Days pos-Transplantation (Tx) are represented in the *x*-axis. The number of par. eq./10 mL of blood is represented in the *y-*axis, in a log-scale. Arrow marks initiation of Benznidazole treatment. ND: not detectable. The line indicates LOQ (1.185 log_10_ par. eq./10 mL) derived from analysis of CL-Brener (TcVI) spiked samples. Discontinued line: parasitic loads in Chacao patients were estimated with Silvio X-10 (TcI) calibration curves.

Figure 4A depicts parasitic loads obtained from peripheral blood samples collected from three patients of G1 at time of diagnosis and after etiological treatment. The tested cases presented *T. cruzi* loads higher than 3 log_10_ par. eq./10 mL of blood at time of diagnosis, becoming undetectable one year after treatment. However, two years after treatment, the qPCR rendered positive results, though with low parasitic loads, indicating that the patients were already in a chronic form because of the treatment failure.

Figure 4B shows parasitic loads from a 42 year-old seronegative man who received kidney transplantation from a seropositive cadaveric donor and became infected. Acute infection by *T. cruzi* was detected 93 days after transplantation by means of qPCR, however it was diagnosed by conventional parasitological (Strout) and serological tests only 121 days after transplantation. Upon conventional diagnosis, treatment with Benznidazole was initiated. Parasitic loads diminished and were non-detectable in the sample collected 159 days after transplantation, persisting non-detectable at least 228 days after transplantation.

## Discussion

### Analytical performance of the qPCR assay

In 2007, an international collaborative study to evaluate current PCR procedures for detection of *T. cruzi* infection was initiated [26]. A high variability was observed among laboratories and methods that used similar DNA extraction procedures and identical primer sequences, confirming that the lack of standardization led to poor reproducibility, precluding the possibility to compare findings among different laboratories. Furthermore, some methods showed an important reduction of the analytical sensitivity when spiked blood samples were tested in comparison to purified parasite DNA, suggesting that the DNA purification step was crucial for the PCR yield. Since most procedures lacked internal amplification controls, discrimination between true and false negative results could often not be assessed. Indeed, PCR cannot be given diagnostic status, before it includes an internal amplification control [32]. Homologous extrinsic controls, as well as heterologous intrinsic and extrinsic controls have been proposed as IACs [30]. The former may give rise to competitive reactions with the target. Heterologous intrinsic controls are often referred as “housekeeping genes” and are conserved fragments of the host's genome that are present naturally in patient specimens in low copy number. These controls are amplified with a different set of primers in the same or a separate reaction vessel. Commonly used intrinsic controls include the genes encoding beta-globin, beta-actin, RNAse P, among others. Depending on the marker chosen and the specimen type, intrinsic controls can be used to establish the presence of cellular material in a clinical specimen. A concern when using intrinsic controls is that the number of human gene copies may be much higher than the target infectious organism copy number and thus have an amplification advantage and not accurately test for inhibition [30]. Furthermore, when analysing blood samples, patients with different blood cell counts will render heterogenic values of the control precluding the possibility to evaluate the yield of DNA extraction, as well as to accurately quantify the target sequence relative to the sample volume. Finally, heterologous extrinsic controls are non-host-derived controls that require primers and probes different from the target. They are added to the sample before DNA preparation and dually serve as extraction and amplification controls. In this context, the latter type of IAC has been used in our multiplex qPCR approach.

In this work, to validate *T. cruzi* qPCR results, the Tukey's boxplot method was carried out using the Ct values of IAC products from all samples tested in every PCR run, in order to detect outlier values of IAC-PCR [32] that would indicate poor DNA yield or inhibition, leading to sub-estimate the parasitic load or to give a false negative result.

Although satellite DNAs belong to the fast-evolving portion of eukaryotic genomes, it has been shown that over 100 satellite units of nine *T. cruzi* strains from different DTUs display almost 100% of nucleotide identity. No DTU-specific consensus motifs have been identified, inferring species-wide conservation [33]. The method was inclusive for all DTUs, though variations in analytical sensitivity were found among parasite stocks belonging to different DTUs, reflecting disparities in gene dosage of their satellite repeats [22], [34], [35]. Interestingly, the qPCR analytical sensitivity was variable among different TcI genotypes too [13], [16], indicating for the first time, heterogeneity in satellite copy numbers within this DTU. In this scenario, trueness of parasitic load measurements should be more accurate if standard curves are built using a strain belonging to the same DTU/genotype of the patient under follow-up. However, this may be unfeasible in clinical practice, because direct typing of parasite DTUs or genotypes from clinical samples is difficult, in particular in chronic Chagas disease patients [9], [10], [13], [36]. Nevertheless, since it has been observed that bloodstream parasite genotypes are persistent during chronic infection [37] or reactivation [36], any parasite stock could be useful as a standard as long as it is included through the whole monitoring of a certain patient or cohort.

*T. rangeli* and *T. cruzi* are found in the same mammalian hosts, sharing triatomine vectors and a significant portion of their antigenic coat, hence *T. rangeli* infections and/or mixed infections by both species may confound the diagnosis. However, *T. cruzi* harbors satellite sequences at a much higher dosage than *T. rangeli* [38]. Moreover, leishmanial infections may lead to serological cross-reaction with *T. cruzi*. The qPCR test was also selective for *T. cruzi* DNA; it did not amplify DNAs from *Leishmania* sp. and amplified *T. rangeli* DNA only at high concentrations (Table 3).

### Application to clinical specimens

Analysis of GEB samples from different groups of individuals, allowed identification of different degrees of qPCR positivity as well as parasitic loads. Among the 16 orally infected cases from G1, 14 were positive in the qPCR test (87.5%) and baseline parasitic loads ranged between 1.69 and 5.23 log_10_ par. eq./10 mL blood, which is compatible with acute infections. Quantitative PCR monitoring is reported for three cases (Figure 4A). This analysis allowed detection of treatment failure two years after the conclusion of treatment. However, at that time parasitic loads were lower than at baseline analysis, which is compatible with the evolution of the infection to the chronic phase. These patients have received a second treatment and are currently under follow-up (Dr Belkisyole Alarcón de Noya, unpublished data).

In chronic Chagas disease cases parasitic loads were low, especially in G2, which was conformed by adult patients from Bolivia. Indeed, many of them gave detectable but non-quantifiable qPCR results (Figure 3). The *T. cruzi* qPCR result of the sample giving an outlier IAC-PCR value (Table 6) was positive (Ct: 32.77, 0.69 log_10_ par. eq./10 mL blood) yet below the LOQ. Then, if a more accurate parasitic load is needed, the DNA extraction and amplification of the same GEB sample should be repeated and the result re-analyzed.

Samples from G3 presented higher degree of PCR positivity and parasitic loads than G2. One difference between both groups is that G3 GEB specimens were boiled before DNA extraction. In fact, many PCR methods using GEB samples incorporated a boiling step before DNA extraction [26]. This was originally designed to enhance sensitivity of procedures based on minicircle DNA amplification [39]. Indeed, incubating samples during 15 minutes favoured fragmentation of minicircle concatemers and distribution of individual minicircles throughout all sample volume, allowing processing of small aliquots (100 µL) with satisfactory sensitivity [39]. In this context, experiments to determine the LOD of the multiplex qPCR assay were carried out from both boiled and non-boiled spiked samples, obtaining slightly higher sensitivity using boiled GEB (0.46 vs 0.70 par. eq./mL, respectively; p = 0.044). So, we can not discard that higher PCR positivity and higher parasitic loads found in G3 chronic cases were partially influenced by the boiling step. However, as the boiling procedure might enhance the risk of cross-contamination among samples, leading to false positive results, we decided to continue the analytical validation of the qPCR using non-boiled spiked samples. Finally, the lower qPCR positivity and parasitic burden of G2 specimens could also be an intrinsic feature of the study population, such as the host genetic background and immunologic status which in turn may play a role in control of parasitic replication. Another factor could be related to the strains involved, though in both countries TcV appears to be the predominant DTU [10], [20], [40].

Among G4 newborns to seropositive mothers, we detected three positive cases, both by qPCR and microhematocrite, which allowed early diagnosis of congenital infection and subsequent treatment with Benznidazole. Thus, clinical sensitivity of qPCR respect to microhematocrite was 100%. The final diagnosis of cases with negative findings by microhematocrite and qPCR will be assessed by means of serological tests at 9 months of age, allowing determination of the qPCR sensitivity respect to final diagnosis. Interestingly, mothers of G4 infected newborns were also qPCR positive (unpublished data), in agreement with the reported correlation between maternal parasitemia and risk of vertical transmission [37], [41].

Cord blood has proven useful for early detection of congenital *T. cruzi* infection, with the advantage of being a non-invasive specimen without volume restrictions [42], [43]. The IAC recovery from G4 peripheral and umbilical cord blood samples showed no significant differences (p = 0,0589). Bern and coworkers observed that qPCR carried out from cord blood samples increased sensitivity for early diagnosis of congenital infection in comparison with conventional parasitological examination [43]. However, risk of contamination with parasite DNA from maternal blood may exist; accordingly the cord must be washed prior to sampling. Standard operative procedures for umbilical cord blood collection are still needed.

The qPCR method was also useful for earlier diagnosis of post-transplant infection in a seronegative receptor of a cadaveric organ explanted from an infected subject. This may allow prompt treatment before the appearance of clinical signs and symptoms of acute disease.

In this work, we have presented multiplex TaqMan qPCR-based results using blood specimens treated with GE, following the criteria used in an international collaborative study [26]. However, in a recent work, TaqMan qPCR strategies targeted to the satellite sequence as well as to minicircle DNA were also satisfactory when tested in fresh-EDTA blood samples and in buffy-coat preparations [44]. Further evaluation of our multiplex qPCR test in different type of biological specimens and conservation conditions will allow its validation for different clinical, experimental and eco-epidemiological settings.

When compared to SYBR Green qPCR strategies [22], the multiplex qPCR assay presents the advantages that it permits simultaneous detection of target DNA and the internal control, allowing identification of reduction in parasitic load or negative findings due to inhibitors or DNA loss; moreover, the TaqMan strategy decreases the likelihood of obtaining false positive results, due to the specificity of TaqMan probes and the multiplex format is less expensive and cumbersome, since only one PCR reaction per sample is needed. It is expected that the use of this qPCR strategy in clinical trials will demonstrate the potential of parasitic loads as surrogate markers of treatment efficacy. Demonstration of cure is up-to-date based on persistent seronegative results after treatment implementation, which in chronic Chagas disease usually takes many years to occur. Especially in these patients, fluctuancy of parasitic loads along lifetime determines that undetectable bloodstream qPCR results can not be taken as indicative of cure. On the contrary, persistence of positive qPCR findings is indeed indicative of treatment failure. In addition, this methodology can offer early diagnosis of infection in cases in which serological methods are not informative, such as transmission by the oral, congenital, transfusional routes or after transplantation with organs from seropositive donors or in events of Chagas disease reactivation due to immunosuppression.

## Supporting Information

Figure S1 **Amplification performance of the IAC in the Multiplex Real Time PCR assay.** Negative GEB samples were spiked with 50 to 800 pg of the linearized IAC plasmid (final concentration after DNA extraction: 0.5 to 8 pg/µl) and DNA extraction was performed in duplicate as well as the PCR assay from each DNA lysate. A. IAC amplification plots obtained using an Applied Biosystems (ABI 7500) device. B. Standard curve and efficiency of IAC amplification.
(TIF)

Table S1 **Estimation of Precision of the qPCR assay.** Precision experiment was carried out on spiked GEB samples with 5, 100 and 10000 par. eq./10 mL, assayed on duplicates during 20 consecutive days, one run per day. Ct: threshold cycle; par. eq./10 mL: parasite equivalents in 10 mL of blood.
(DOC)
