# Supplementary figures and images for: Analytical Performance of a Multiplex Real-Time PCR Assay Using TaqMan Probes for Quantification of Trypanosoma cruzi Satellite DNA in Blood Samples
Source: PLoS Negl Trop Dis. 2013 Jan 17;7(1):e2000. doi: 10.1371/journal.pntd.0002000 (PMC3547845; doi:10.1371/journal.pntd.0002000)

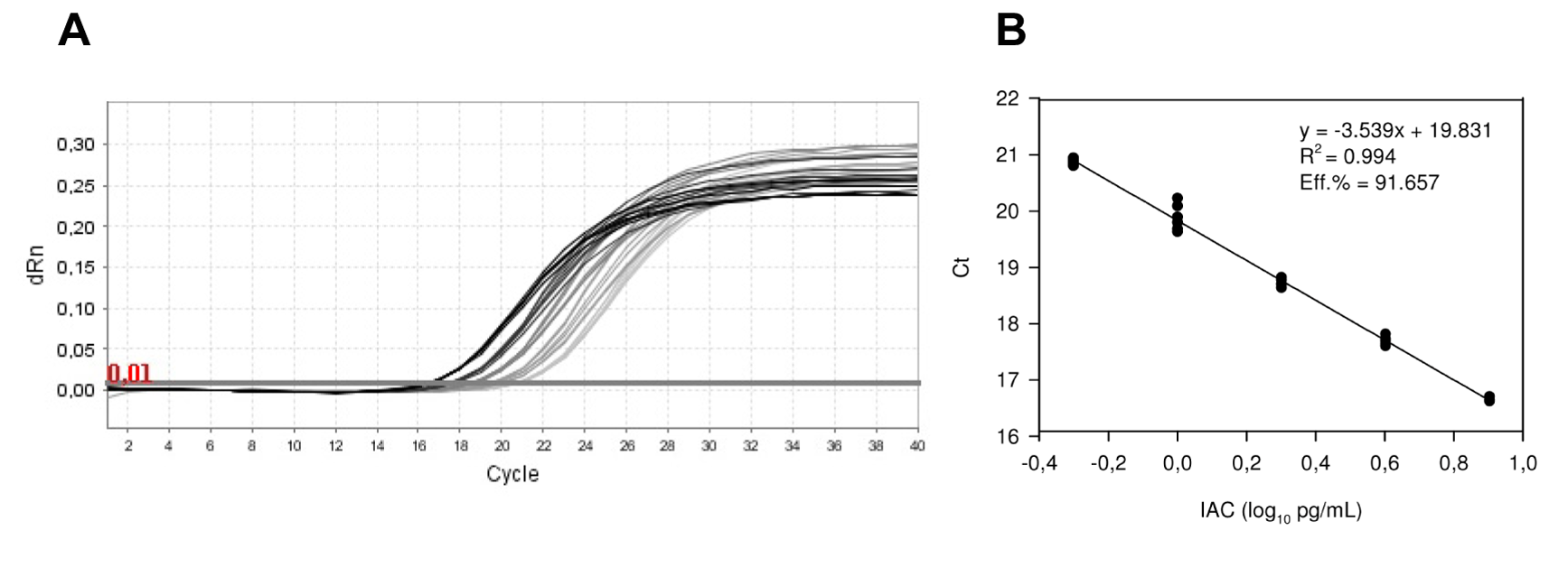

Supplement: Figure S1 — Amplification performance of the IAC in the Multiplex Real Time PCR assay. Negative GEB samples were spiked with 50 to 800 pg of the linearized IAC plasmid (final concentration after DNA extraction: 0.5 to 8 pg/µl) and DNA extraction was performed in duplicate as well as the PCR assay from each DNA lysate. A. IAC amplification plots obtained using an Applied Biosystems (ABI 7500) device. B. Standard curve and efficiency of IAC amplification. (TIF) [file pntd.0002000.s001.tif]
