# Supplementary material for: Analytical Performance of a Multiplex Real-Time PCR Assay Using TaqMan Probes for Quantification of Trypanosoma cruzi Satellite DNA in Blood Samples
Source: PLoS Negl Trop Dis. 2013 Jan 17;7(1):e2000. doi: 10.1371/journal.pntd.0002000 (PMC3547845; doi:10.1371/journal.pntd.0002000)
Supplement: Table S1 — Estimation of Precision of the qPCR assay. Precision experiment was carried out on spiked GEB samples with 5, 100 and 10000 par. eq./10 mL, assayed on duplicates during 20 consecutive days, one run per day. Ct: threshold cycle; par. eq./10 mL: parasite equivalents in 10 mL of blood. (DOC) [file pntd.0002000.s002.doc]

**TABLE S1. Estimation of Precision of the qPCR assay**

|  | **5 par. eq./ 10 mL** | | | **100 par. eq./ 10 mL** | | | **10000 par. eq./ 10 mL** | | |
| --- | --- | --- | --- | --- | --- | --- | --- | --- | --- |
| **Day** | **Ct** | **par. eq./ 10 mL** | **log_10_ par. eq. /10 mL** | **Ct** | **par. eq./ 10 mL** | **log_10_ par. eq. / 10 mL** | **Ct** | **par. eq./ 10 mL** | **log_10_ par. eq. /10 mL** |
| **1** | 29.56 | 46.9 | 1.67 | 26.58 | 353 | 2.55 | 19.99 | 30100 | 4.48 |
|  | 32.21 | 7.9 | 0.90 | 28.04 | 132 | 2.12 | 20.09 | 28300 | 4.45 |
| **2** | 32.77 | 5.4 | 0.73 | 26.20 | 454 | 2.66 | 19.79 | 34500 | 4.54 |
|  | 32.28 | 7.5 | 0.87 | 26.55 | 359 | 2.56 | 19.82 | 34000 | 4.53 |
| **3** | 30.67 | 22.2 | 1.35 | 26.93 | 278 | 2.44 | 20.22 | 25900 | 4.41 |
|  | 32.12 | 8.3 | 0.92 | 26.51 | 368 | 2.57 | 19.77 | 34900 | 4.54 |
| **4** | 30.54 | 24.2 | 1.38 | 26.36 | 408 | 2.61 | 19.90 | 32000 | 4.51 |
|  | 30.84 | 19.7 | 1.29 | 26.58 | 352 | 2.55 | 20.02 | 29600 | 4.47 |
| **5** | 31.99 | 9.1 | 0.96 | 26.40 | 398 | 2.60 | 19.86 | 32900 | 4.52 |
|  | 30.77 | 20.7 | 1.32 | 26.62 | 342 | 2.53 | 19.86 | 32900 | 4.52 |
| **6** | 36.40 | 0.5 | -0.34 | 26.52 | 367 | 2.56 | 19.73 | 35900 | 4.56 |
|  | 33.44 | 3.4 | 0.53 | 26.30 | 424 | 2.63 | 19.83 | 33600 | 4.53 |
| **7** | 30.72 | 21.5 | 1.33 | 26.85 | 293 | 2.47 | 19.97 | 30700 | 4.49 |
|  | 31.10 | 16.6 | 1.22 | 26.54 | 360 | 2.56 | 19.76 | 35300 | 4.55 |
| **8** | 31.57 | 12.0 | 1.08 | 26.96 | 273 | 2.44 | 19.34 | 46800 | 4.67 |
|  | 29.94 | 36.4 | 1.56 | 26.69 | 327 | 2.51 | 19.83 | 33500 | 4.53 |
| **9** | 29.73 | 41.9 | 1.62 | 26.70 | 324 | 2.51 | 20.11 | 27800 | 4.44 |
|  | 32.27 | 7.5 | 0.88 | 27.00 | 265 | 2.42 | 20.06 | 28700 | 4.46 |
| **10** | 30.29 | 28.7 | 1.46 | 27.01 | 263 | 2.42 | 20.18 | 26600 | 4.42 |
|  | 31.99 | 9.1 | 0.96 | 26.88 | 287 | 2.46 | 20.25 | 25400 | 4.40 |
| **11** | 30.57 | 23.7 | 1.37 | 26.30 | 424 | 2.63 | 19.73 | 36100 | 4.56 |
|  | 29.85 | 38.7 | 1.59 | 26.73 | 319 | 2.50 | 19.97 | 30600 | 4.49 |
| **12** | 32.20 | 7.9 | 0.90 | 26.21 | 453 | 2.66 | 19.67 | 37400 | 4.57 |
|  | 30.61 | 23.0 | 1.36 | 26.31 | 422 | 2.63 | 20.10 | 28100 | 4.45 |
| **13** | 30.85 | 19.6 | 1.29 | 26.57 | 354 | 2.55 | 20.04 | 29200 | 4.47 |
|  | 30.14 | 31.6 | 1.50 | 26.78 | 307 | 2.49 | 20.03 | 29500 | 4.47 |
| **14** | 28.61 | 89.5 | 1.95 | 26.34 | 412 | 2.61 | 19.47 | 43000 | 4.63 |
|  | 31.59 | 11.9 | 1.08 | 26.45 | 385 | 2.59 | 19.91 | 31900 | 4.50 |
| **15** | 31.18 | 15.7 | 1.20 | 26.09 | 488 | 2.69 | 19.71 | 36400 | 4.56 |
|  | 31.03 | 17.4 | 1.24 | 26.35 | 412 | 2.61 | 19.76 | 35200 | 4.55 |
| **16** | 31.09 | 16.7 | 1.22 | 26.32 | 420 | 2.62 | 19.71 | 36500 | 4.56 |
|  | 30.21 | 30.2 | 1.48 | 26.51 | 369 | 2.57 | 19.96 | 30700 | 4.49 |
| **17** | 32.57 | 6.2 | 0.79 | 26.81 | 302 | 2.48 | 19.71 | 36500 | 4.56 |
|  | 30.94 | 18.5 | 1.27 | 27.20 | 231 | 2.36 | 19.47 | 42800 | 4.63 |
| **18** | 29.46 | 50.3 | 1.70 | 26.47 | 379 | 2.58 | 19.61 | 39000 | 4.59 |
|  | 28.93 | 71.6 | 1.85 | 26.43 | 389 | 2.59 | 19.75 | 35600 | 4.55 |
| **19** | 31.91 | 9.6 | 0.98 | 26.39 | 401 | 2.60 | 19.84 | 33300 | 4.52 |
|  | 31.42 | 13.3 | 1.12 | 26.06 | 499 | 2.70 | 19.84 | 33400 | 4.52 |
| **20** | 32.89 | 4.9 | 0.69 | 26.23 | 445 | 2.65 | 19.84 | 33400 | 4.52 |
|  | 31.81 | 10.3 | 1.01 | 26.11 | 484 | 2.68 | 20.21 | 26000 | 4.41 |
